# Fertility quality of life (FertiQoL) among Chinese women undergoing frozen embryo transfer

**DOI:** 10.1186/s12905-021-01325-1

**Published:** 2021-04-24

**Authors:** Donghong Song, Xue Li, Min Yang, Na Wang, Yang Zhao, Siyu Diao, Xi Zhang, Xuemei Gou, Xiu Zhu

**Affiliations:** 1 Reproductive Medicine Center, Peking University Third Hospital, Beijing, China; 2 Peking University School of Nursing, Beijing, China; 3 School of Nursing and Midwifery, Griffith University, Gold Coast City, Australia

**Keywords:** Infertility, Quality of life, Infertility-related stress, Anxiety

## Abstract

**Background:**

Women undergoing infertility treatment have poor quality of life. This may cause them to withdraw from or refuse treatment. Women undergoing frozen embryo transfer have a treatment interval. The aim of this study was to investigate the status quo of the fertility quality of life in women undergoing frozen embryo transfer and analyse its predictors.

**Methods:**

A cross-sectional survey was conducted from August 2019 to August 2020 among women undergoing frozen embryo transfer in a tertiary hospital reproductive centre in Beijing, China. The survey collected demographic characteristics and treatment data and included the fertility problem inventory, the fertility quality of life scale (FertiQoL) and the state-trait anxiety scale. Multiple linear stepwise regression was used to explore the predictors of fertility quality of life.

**Results:**

In total, 1062 women completed the survey. Participants reported that they had high levels of fertility-related stress and anxiety during treatment. They also had lower fertility-related quality of life, and the Treatment FertiQoL scored the lowest. The regression results showed that social concern, trait anxiety, duration of treatment and age were risk factors for diminished fertility quality of life.

**Conclusion:**

Chinese women undergoing frozen embryo transfer have relatively poor quality of life. The potential predictors of fertility quality of life include social concern, trait anxiety, duration of treatment and age.

**Supplementary Information:**

The online version contains supplementary material available at 10.1186/s12905-021-01325-1.

## Background

Infertility has been newly defined as a disease characterized by the failure to establish a clinical pregnancy after 12 months of regular, unprotected sexual intercourse or due to impairment of a person's capacity to reproduce either as an individual or with his/her partner [1]. Infertility is not just a reproductive dysfunction; it also leads to psychological problems. In vitro fertilization (IVF) is currently an important means of treatment for infertility. However, IVF has a long treatment period involving multiple injections, blood sample collection and ultrasound scanning, and it may cause complications such as ovarian hyperstimulation syndrome (OHSS). The treatment process and uncertain results may increase the psychological burden of patients [2, 3]. In addition, due to China's traditional culture, women receiving treatment in this context may have to endure prejudice from family and society [4].

Quality of life is an individual's perception of his or her place in life in the context of the culture and value system in which he or she lives [5]. Previous studies have shown that infertility and its treatment process may lead to psychological stress [5], hence threatening the quality of life of patients [6]. Psychological pressure and impaired quality of life can also lead to premature withdrawal from treatment [7] or reluctance to receive treatment [8], with consequences for patients’ treatment outcomes. Previous studies suggest that, compared with fertile women, infertile women tend to have a lower level of quality of life and greater psychological burden. The potential factors found in previous studies to influence quality of life include patients’ personal factors (education level, employment situation, income, etc.), social relations (husband-wife relations, family relations, etc.), psychological status, cause of infertility and treatment process [2, 9, 10]. Studies from China have shown that predictors of patients’ quality of life include the phase of infertility treatment [3, 11], abortion history [12], anxiety [13], fertility stress [14], the shame of not having a child [15], etc.

With the development of assisted reproductive technology, the pregnancy rate for frozen embryo transfer has increased [16]. Single embryo transfer is increasingly used to reduce multiple pregnancies and increase the opportunities for additional frozen embryo transfer and the cumulative pregnancy rate per egg retrieval [17]. In addition, frozen embryo transfer can decrease the risk of OHSS and achieve better embryo-endometrium synchronization through the endometrial preparation cycle [16], hence avoiding adverse patient or medical treatment factors [18]. A study of women undergoing fresh embryo transfer in China showed that the level of their state anxiety fluctuated from the beginning of the treatment cycle to the embryo transfer day. Women experienced the most anxiety at the beginning, followed by the embryo transfer day, and the least anxiety on the day of oocyte retrieval. This result indicates that the psychological state of women undergoing IVF differs across phases [19]. However, women undergoing frozen embryo transfer face a longer wait time between the oocyte retrieval day and the transfer day, which may affect their psychological well-being and quality of life. Therefore, it is meaningful to investigate this group’s psychological state and quality of life in the context of an increasing frozen embryo transfer rate. There is limited evidence on the psychological status and quality of life of women undergoing frozen embryo transfer. To our knowledge, this is the first article to address the quality of life of frozen embryo transfer patients. Additionally, sociocultural differences impact women’s responses when accessing embryo transfer services. Our study provided data from a Chinese population and will therefore add current evidence from women in a non-Western context. This study will investigate the fertility quality of life of Chinese women undergoing frozen embryo transfer and analyse the predictors to provide a basis for comprehensive intervention.

## Methods

### Study design and participants

A cross-sectional survey was conducted among women undergoing frozen embryo transfer from September 2019 to September 2020 in a tertiary hospital reproductive centre in Beijing, China. The questionnaire was completed by the women themselves under the guidance of investigators. To ensure standardization, we provided unified training for the investigators to ensure that each item was understood consistently.

The inclusion criteria were as follows: (1) women who were diagnosed with infertility according to WHO standards and intended to be assisted by frozen embryo transfer; (2) women who were able to read and write independently; and (3) women who volunteered and signed the informed consent form. The exclusion criteria were as follows: (1) a history of severe mental health problems or recent major life events; (2) unable to cooperate with the investigation.

### Study instruments

#### Basic information questionnaire

This questionnaire was constructed by the research team after reviewing the prior literature. It includes two parts: demographic characteristics and treatment data. Demographic characteristics included women’s age, residence, education level, monthly household income, employment status, marital status and parity. Treatment data included duration of infertility (years), duration of treatment (years), cause of infertility (female factor infertility, male factor infertility, combined factor infertility, or unknown), type of infertility (secondary, idiopathic), previous IVF experience, number of embryo transfers, abortion history, treatment history (any previous treatment related to infertility except IVF, such as oral ovulation induction), recipient of treatment (who received the above infertility-related treatment: female, male or both), and whether diagnosed with gynaecological diseases before (yes or no).

#### Fertility problem inventory (FPI)

The FPI was designed by Newton CR [20] to evaluate fertility-related problems in infertile patients and has been widely used in infertility research. The Chinese version was validated for the clinical evaluation of infertility in China [21], with a Cronbach's α of 0.81. It includes 46 items to evaluate fertility problems in five domains: social concern (10 items), relationship concern (10 items), sexual concern (8 items), the need for parenthood (8 items), and rejection of a child-free lifestyle (10 items). The scale uses a 6-point Likert scoring standard, and the scores of each item range from 1 to 6. "1" means "totally disagree", and "6" means "totally agree". The social concern, relationship concern, and need for parenthood dimension scores range from 10 to 60. The sexual concern and rejection of a child-free lifestyle dimension scores range from 8 to 48. The total score ranges from 46 to 276. A higher score indicates higher fertility-related pressure.

#### Fertility quality of life (FertiQoL)

This scale is a self-rating questionnaire specifically designed to assess the quality of life of infertile patients. It has good reliability and effectiveness in many countries. The Cronbach’s α of the Chinese version of the FertiQoL is 0.925 [14]. The FertiQoL scale contains two modules: a Core FertiQoL module and an optional Treatment FertiQoL module. The Core FertiQoL module is divided into four fields: emotional (6 items), mind–body (6 items), relationship (6 items) and social (6 items). The optional Treatment FertiQoL module is divided into two fields, namely, environment (6 items) and tolerability of infertility treatment (4 items). The score of each item is 0–4 points, and the total scale and subscale scores can be converted to 0–100-point scales. The higher the score, the higher the quality of life is.

#### State-trait anxiety inventory (STAI)

The STAI is divided into two subscales to evaluate two different anxiety types, and each subscale (the S-AI and T-AI) contains 20 items. State anxiety refers to a temporary emotional state characterized by subjective tension, and its intensity may change with time. Trait anxiety refers to a relatively stable behavioural tendency of individuals to respond to a wide range of threatening stimuli. The reliability and effectiveness of the STAI have been verified in different groups, and its Cronbach's α ranges from 0.86 to 0.95 [22]. The Chinese version’s Cronbach’s α was 0.90 [23]. The questionnaire is completed by self-assessment and can be used for individual or group tests. The STAI adopts a 4-point scoring standard, as follows: S-AI: 1 = not at all, 2 = some, 3 = moderate, 4 = very obvious; T-AI: 1 = almost none, 2 = some, 3 = often, 4 = almost always. Positively expressed items are reverse scored when analysing the data; for example, "1" is labelled "4" and “4” is labelled “1”. The score of each sub-test ranges from 20 to 80. A higher score indicates a more serious level of anxiety.

### Data analysis

Excel was used for data entry, and SPSS 25.0 (IBM Company, Chicago, IL, USA) was used for data analysis. Descriptive statistics are expressed in terms of frequency, percentage, mean, and standard deviation. The women’s demographic characteristics, disease and treatment history, and S-AI, T-AI and FPI scores were the independent variables and the total FertiQoL score was the dependent variable in the multiple linear stepwise regression analysis. A value of *p* < 0.05 was considered to be statistically significant.

## Results

### Demographic characteristics of participants

We distributed a total of 1136 questionnaires to women undergoing frozen embryo transfer, and 1062 valid questionnaires were obtained, for an effective recovery rate of 93.5%. The average age of the participants was 33.08 (SD = 4.23), and the average length of marriage was 6.20 (SD = 3.58) years. More than half of the women were employed, and most were in their first marriage. The specific results are shown in Table 1.

**Table 1:** Demographic characteristics of the patients (N = 1062)

Item		n	%	Item		n	%
Residence	City	837	78.8	Marital status	First marriage	935	88.0
	Countryside	217	20.4		Remarriage	88	8.3
	Missing	8	0.8		Missing	39	3.7
Employment status	Unemployed	335	31.5	Monthly household income (CNY)	< 4000	141	13.4
	Employed	717	67.6		4000–7000	176	16.8
	Missing	10	0.9		7000–10,000	228	21.7
Education level	Below college	479	45.1		> 10,000	505	48.1
	College or university	417	39.3		Missing	12	1.0
	Master’s and above	166	15.6	Parity	0	918	86.4
					≥ 1	114	10.7
					Missing	30	2.9

### Treatment data of participants

Among the surveyed women, the infertility duration varied from less than 3 years to more than 10 years. The causes of infertility were mainly female factor (36.3%), and more than half of the women had experience with IVF. Further treatment information is shown in Table 2.

**Table 2:** Treatment data of the patients (N = 1062)

Item		n	%	Item		n	%
Duration of infertility (years)	< 3	268	25.5	Abortion history	0	581	50
	3–5	303	26.1		1	258	22.2
	5–10	389	33.5		≥ 2	207	17.9
	> 10	89	7.7		Missing	16	1.5
	Missing	13	1.2	Duration of treatment (years)	1	328	28.2
Cause of infertility	Female factor	386	36.3		2	246	21.2
	Male factor	207	19.5		3–5	308	26.5
	Combined	172	16.2		> 5	157	13.5
	Unknown	250	23.5		Missing	23	2.1
	Missing	47	4.4	Treatment history	Yes	541	50.9
Type of infertility	Secondary	316	27.2		No	475	44.7
	Idiopathic	243	20.9		Missing	46	4.4
	Missing	503	47.4	Treatment recipient	Female	73	6.9
Previous IVF experience	Yes	628	59.1		Male	362	34.1
	No	412	38.8		Both	224	21.1
	Missing	22	2.1		Missing	403	37.9
Number of embryo transfers	1	333	31.4	Whether diagnosed with gynaecological diseases before	Yes	306	28.8
	2	340	32.0		No	682	64.2
	> 3	348	32.8		Missing	74	7.0
	Missing	41	3.9				

### Scale scores

In this study, the mean total FPI score was 136.5 (SD = 29.4). The mean social concern dimension score was 27.1 (SD = 8.5). The mean relationship concern dimension score was 24.6 (SD = 7.7). The mean need for parenthood dimension score was 37.9 (SD = 8.3). The mean rejection of a child-free lifestyle dimension score was 26.7 (SD = 7.2), and the mean sexual concern dimension score was 18.4 (SD = 6.9). The specific scores are shown in Additional file 1.

The mean S-AI and T-AI scores were 41.9 (SD = 10.7) and 42.0 (SD = 10.1), respectively. The specific scores are shown in Additional file 1.

According to the scoring standard of the FertiQoL, the total FertiQoL score, the Core FertiQoL score and the Treatment FertiQoL score were calculated. The specific scores are shown in Table 3. The mean total FertiQoL score was 64.5 (SD = 14.1); the mean Core FertiQoL score was 66.3 (SD = 16.0), and the mean Treatment FertiQoL score was 57.9 (SD = 19.8).

**Table 3:** Patients’ Fertility Quality of Life (N = 1062)

Item	n	Minimum	Maximum	Mean	SD
Total FertiQoL score	989	18.4	100.00	64.5	14.1
Core FertiQoL	1054	12.5	100.00	66.3	16.0
Emotional	1059	0.00	100.00	64.1	19.9
Mind/body	1051	0.00	100.00	63.4	22.8
Relational	1057	16.7	100.00	67.9	14.8
Social	1042	8.3	100.00	69.9	16.9
Treatment FertiQoL	1057	20.0	100.00	59.9	13.3
Environment	1010	16.7	100.00	61.2	14.0
Tolerability	1007	0.00	100.00	57.9	19.8

### FertiQoL predictors

According to the regression results (Table 4), social concern (SB =  − 0.439), trait anxiety (SB =  − 0.290), duration of treatment (SB =  − 0.128) and age (SB =  − 0.114) entered the regression equation (R^2^ = 42.8%), which means that these variables were risk factors for poor fertility quality of life. Social concern (SB =  − 0.439) was the strongest predictor of fertility quality of life, followed by trait anxiety (SB =  − 0.290).

**Table 4:** Multiple linear stepwise regression results for fertility quality of life

Independent variable	SB	95%CI		t	*p*
		Lower	Upper		
Social concern	− 0.439	− 0.934	− 0.513	− 6.781	< 0.001
Trait anxiety	− 0.290	− 0.591	− 0.231	− 4.510	< 0.001
Duration of treatment	− 0.128	− 3.234	− 0.218	− 2.258	0.025
Age	− 0.114	− 0.743	− 0.003	− 1.988	0.048

SB indicates standard beta

## Discussion

The mean total FertiQoL score of women undergoing frozen embryo transfer in this study was 64.5 ± 14.1; the mean Core FertiQoL score was 66.3 ± 16.0 out of 100. To our knowledge, there are no other studies investigating fertility quality of life in frozen embryo transfer populations. Therefore, we can only refer to the results of IVF population (including both fresh and frozen embryo transfer patients) studies. The mean FertiQoL score in this study was similar to that in other studies conducted in China. For example, the mean total FertiQoL and the mean Core FertiQoL scores of women undergoing IVF in Shanxi Province (a mountainous province in central China) were 63.6 ± 13.72 and 65.10 ± 16.10, respectively [14], and the mean Core FertiQoL scores for women undergoing IVF were 64.54 ± 16.90 in Shenyang [23] and 61.8 ± 18.0 in Lanzhou [15]. However, these scores are lower than those seen in a mixed populations of fresh and frozen embryo transfer patients in developed countries. In the USA, for example, the mean Core FertiQoL score of women undergoing IVF was 72.30 ± 14.80 [14]. Regarding IVF technology, there was little difference between China and developed countries, so cultural differences and psychosocial issues may be more important.

In this study, the mean Treatment FertiQoL score was 59.9 ± 13.3, with a mean environment score of 61.2 ± 14.0 and a mean tolerability score of 57.9 ± 19.8. Compared with the results obtained for the IVF population in Lanzhou city (Environment: 64.9 ± 13.0; Tolerability: 54.4 ± 19.4) [11] in China, the environment score for women undergoing frozen embryo transfer was poor, though the mean tolerability score was better. This may be because the current study was conducted in a well-known metropolitan reproductive medicine centre in China, and many patients have to spend a considerable amount of time waiting in line to receive treatment. It may also be due to women undergoing frozen embryo transfer facing longer wait times at home, and women may feel they do not receive adequate attention and psychological support from medical staff, affecting their psychological well-being and quality of life. The tolerability score may be due to the fact that women undergoing frozen embryo transfer have had previous treatment experiences, such as ovulation induction and egg retrieval, making them more tolerant of treatment.

The results showed that social concern was the strongest predictor of fertility quality of life. Some studies reported a negative correlation between fertility-related stress and quality of life [24], especially in the case of social stress [25]. In developing countries, having no children may be a threat to personal values, social security, status, gender identity, and family origin [3]. Society often blames women for infertility and even stigmatizes them [15], so women may experience more social prejudice. Studies have confirmed that resilience can help individuals cope with stress [14], while mindfulness therapy and cognitive behavioural therapy can help patients build resilience [26, 27]. Interventions based on psychological theories could be used among women receiving IVF services to improve their quality of life.

Trait anxiety was also an important predictor of the FertiQoL score, and state anxiety was not included in the regression equation, which means that trait anxiety affects quality of life more. In this study, the mean state anxiety score was 41.9 ± 10.7, and the mean trait anxiety score was 42.0 ± 10.1. A study from China conducted in the same hospital reported that the mean state anxiety and the mean trait anxiety scores of women who received fresh embryo transfer were 39.8 ± 11.9 and 40.0 ± 10.5, respectively [19]. Compared with women undergoing fresh embryo transfer, the women who received frozen embryo transfer reported greater anxiety when receiving treatment (41.9 ± 10.7 vs. 39.8 ± 11.9, t = 2.419, *p* = 0.019). This result indicates that the treatment interval may exacerbates the anxiety of women undergoing embryo transfer. More studies are needed to explore whether there is a difference between frozen and fresh embryo transfer in psychological impact.

In addition, duration of treatment (SB =  − 0.128) and age (SB =  − 0.114) were also predictors of fertility quality of life. The longer the treatment time is, the lower the quality of life of patients [15]. Long years of treatment means that there may be more failures and frustration [28], and women may develop self-doubt and experience greater pressure, which threaten their quality of life. In this study, young women had better quality of life scores. Another study from China supports this conclusion [29]. With increasing age, women's ovarian function gradually decreases, and their hormone levels change. As a result, the success rate of IVF decreases, and women face greater fertility pressure. In contrast, some studies in other countries have shown that the quality of life of young women is worse [30, 31] and that older women gradually adapt to infertility and have the ability to cope. Other studies have shown that there is no relationship between age and quality of life [32, 33]. These differences in the research results may be related to the cultural context and research tools used. Therefore, we should also pay attention to the quality of life of older patients and those with long treatment times.

## Strengths and Limitations

The strengths of this study included its use of assessment tools verified and used in many countries. In addition, this study was the first to take Chinese women undergoing frozen embryo transfer treatment as the research objects, and the quality of life status and predictors in this group were investigated. Finally, this study had a relatively large sample size, and the results were representative. However, there were still some limitations in this study. First, it was a cross-sectional study, so it was challenging to deduce a clear causal relationship. Second, the study subjects included only women undergoing frozen embryo transfer and did not include women undergoing fresh embryo transfer, so it was impossible to make fertility quality of life comparisons across medical conditions. Future research is needed to compare these two groups.

## Conclusion

Similar to the findings of other studies conducted in China on IVF populations, women undergoing frozen embryo transfer have relatively poor quality of life. The potential predictors of fertility quality of life include social concern, trait anxiety, duration of treatment and age. These predictors can help us identify high-risk groups, and perhaps we can take measures to intervene in and improve their quality of life in the future.

## Declaration of Helsinki

The research program conformed to Helsinki Declaration and guidelines of institution.

## Supplementary Information

**Additional file 1**. The specific scores of FPI and STAI scales.

**Additional file 2**. Questionnaire of patients’ quality of life.

## Data Availability

The datasets used and analysed during the current study available from the corresponding author on reasonable request.
