# Supplementary material for: Fertility quality of life (FertiQoL) among Chinese women undergoing frozen embryo transfer
Source: BMC Womens Health. 2021 Apr 24;21:177. doi: 10.1186/s12905-021-01325-1 (PMC8070327; doi:10.1186/s12905-021-01325-1)
Supplement: Supplementary file 1 — Additional file 1. The specific scores of FPI and STAI scales. [file 12905_2021_1325_MOESM1_ESM.docx]

**Additional file 1**

The specific scores of FPI and STAI scales are shown in Stable1.

Stable1.Patient's score of FPI and STAI(N=1062)

| Scale | Item | n | Minimum | Maximum | Mean | SD |
| --- | --- | --- | --- | --- | --- | --- |
| FPI | the total score of FPI | 905 | 63.0 | 233.0 | 136.5 | 29.4 |
|  | Social concern | 1014 | 10.0 | 54.0 | 27.1 | 8.5 |
|  | Relationship concern | 1021 | 10.0 | 54.0 | 24.6 | 7.7 |
|  | The need for parenthood | 1007 | 13.0 | 60.0 | 37.9 | 8.3 |
|  | Sexual concern | 1022 | 8.0 | 39.0 | 18.4 | 6.9 |
|  | Rejection of a child-free lifestyle | 1018 | 8.0 | 48.0 | 26.7 | 7.2 |
| TATI | State anxiety | 951 | 20.0 | 78.0 | 41.9 | 10.7 |
|  | Trait anxiet | 943 | 20.0 | 75.0 | 42.0 | 10.1 |
