# Supplementary material for: Fertility quality of life (FertiQoL) among Chinese women undergoing frozen embryo transfer
Source: BMC Womens Health. 2021 Apr 24;21:177. doi: 10.1186/s12905-021-01325-1 (PMC8070327; doi:10.1186/s12905-021-01325-1)
Supplement: Supplementary file 2 — Additional file 2. Questionnaire of patients’ quality of life. [file 12905_2021_1325_MOESM2_ESM.doc]

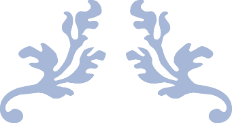


**Questionnaire of patients' quality of life**

Donghong Song1, Xue Li 1,Min Yang2, Na Wang3 ,Yang Zhao 1, Siyu Diao 1, Xi Zhang 1, Xuemei Gou 1, Xiu Zhu 2*


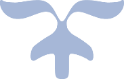


**CONTENT**

[Questionnaire on the basic situation of infertile patients 1](#__RefHeading___Toc58964042)

[Fertility-related stress questionnaire (FPI) 2](#__RefHeading___Toc58964043)

[Quality of life questionnaire 8](#__RefHeading___Toc58964044)

[State-Trait Anxiety Inventory(STAI) 11](#__RefHeading___Toc58964045)

#### Questionnaire on the basic situation of infertile patients

**The following questions are about your basic situation. Please tick "√" or fill in one by one.**

**The basic information of patients:**

**1. gender: □ male □ female**

**2. age: _ _ _ _**

**3. Residence: □ Urban □ Rural**

**4. Education level: □ below junior high school □ high school □ technical secondary school □ junior college □ undergraduate □ graduate student or above**

**5. Employment status: □ unemployed □ on-the-job**

**6. Occupation: □ None □ Worker □ Farmer □ Staff □ Cadre □ Teacher □ Doctor □ Individual □ Others**

**7. Family monthly income: □ less than 4,000 yuan □ 4,000-7,000 yuan □ 7,000-10,000yuan □ more than 10,000 yuan**

**The fertility of patients:**

**1. Marriage time: _ _ _ _ _ years □ First marriage □ Remarriage**

**2. Infertility time: □ less than 2 years □2-3years □ 3-4years □ 5-10 years □ more than 10 years**

**3. Birth history: □ No □ One child □ Two children**

**4. Abortion frequency: □ none □1 □2 □ more than 3 times**

**5. Cause analysis of infertility**

**(1)□ Women's factor □ Men's factor □ Both parties □ The reason is unknown**

**(2)□ Primary infertility □ Secondary infertility**

**6. Past gynecology: □ Yes □ No.**

**The treatment of infertility:**

**1. Treatment period: □1 year □2 years □ 3 ~ years □ more than 5 years**

**2. Previous treatment history: □ Yes □ No.**

**(1) if yes, the recipients: □ woman □ man □ both parties**

**Past treatment methods: □ Chinese medicine therapy □ Western medicine therapy □ Integrated Chinese and Western medicine therapy □ Others**

**3. The current method of assisting pregnancy is □ IVF □ artificial insemination**

**4. History of assisted reproductive technology: □ Yes □ No**

**5. Current pregnancy assisting cycle: □ 1st □ 2nd □ 3rd ~ 5th □ more than 5 times**

#### Fertility-related stress questionnaire (FPI)

**Guidelines for filling out the form: The following statements are different views on fertility. To what extent do you agree or disagree with this view, just tick the answer.**

**Items totally disagree, basically disagree, slightly disagree, slightly agree, basically agree, completely agree.**

| Item | totally disagree | basically disagree | slightly disagree | slightly agree | basically agree | totally agree |
| --- | --- | --- | --- | --- | --- | --- |
| 1.    Couples without children have the same happiness as couples with children. |  |  |  |  |  |  |
| 2. Pregnancy and childbirth are the two most important events in a couple's relationship |  |  |  |  |  |  |
| 3. I lost my enjoyment of sex because of fertility problems |  |  |  |  |  |  |
| 4. I think I am as attractive to my spouse as before |  |  |  |  |  |  |
| For me, being a parent is far more important than a satisfactory career |  |  |  |  |  |  |
| 6. My marriage needs children (or another child) |  |  |  |  |  |  |
| 7. I don't feel any different from other homosexuals |  |  |  |  |  |  |
| 8. If you don't have children, you will feel like a real adult. |  |  |  |  |  |  |
| 9. I won't be bothered when asked questions about children |  |  |  |  |  |  |
| 10. a future without children (or another child) will scare me |  |  |  |  |  |  |
| 11. I can't express my feelings to my partner because doing so will make him/her feel uncomfortable. |  |  |  |  |  |  |
| 12. Family members will not treat us differently because of this |  |  |  |  |  |  |
| 13. I feel that I have failed in sex |  |  |  |  |  |  |
| 14. holidays are a particularly difficult time for me |  |  |  |  |  |  |
| 15. I can find the benefits of having no children (or having one more child) |  |  |  |  |  |  |
| 16. my partner can't understand the influence of fertility problem on me |  |  |  |  |  |  |
| 17. When I have sex, my only thought is to have children (or have one more child) |  |  |  |  |  |  |
| 18. My spouse and I can handle our infertility problem well |  |  |  |  |  |  |
| 19. I feel empty because of our fertility problem |  |  |  |  |  |  |
| 20. I can imagine a happy life without children (or one less child) |  |  |  |  |  |  |
| 21. My spouse's reaction to this question bothers me |  |  |  |  |  |  |
| 22. Because I don't want to experience disappointment again, I find it difficult to have sex. |  |  |  |  |  |  |
| 23. having one child (or one more child) is not the focus of my life |  |  |  |  |  |  |
| 24. My spouse is quite disappointed with me |  |  |  |  |  |  |
| 25. I often dream of having a child (or having one more child) |  |  |  |  |  |  |
| 26. My spouse and I can talk more openly about our fertility |  |  |  |  |  |  |
| 27. Family gatherings are very uncomfortable for me |  |  |  |  |  |  |
| 28. having no children (or having one more child) will give me time to do other things that satisfy me |  |  |  |  |  |  |
| 29. I always thought that I was born to be a parent |  |  |  |  |  |  |
| 30. I can't help but compare with friends with children |  |  |  |  |  |  |
| 31. having children (or having one more child) is not necessary for my happiness |  |  |  |  |  |  |
| 32. if we miss sex on an important day, I will be very angry |  |  |  |  |  |  |
| 33. I can't imagine that my spouse and I will be separated because of infertility |  |  |  |  |  |  |
| 34. I have wanted to be a parent since I can remember. |  |  |  |  |  |  |
| 35. Compared with other friends with children, we still have a lot in common |  |  |  |  |  |  |
| 36. When we try to talk about fertility, it always seems to cause controversy |  |  |  |  |  |  |
| 37. Sometimes I feel so stressed that sex becomes difficult. |  |  |  |  |  |  |
| 38. even without children, our relationship is long and pleasant |  |  |  |  |  |  |
| 39. I find it difficult to get along with friends with children |  |  |  |  |  |  |
| 40. I feel lost when I see a family with children |  |  |  |  |  |  |
| 41. I feel a little free without children |  |  |  |  |  |  |
| 42. I will make any effort for the new child (or one more child) |  |  |  |  |  |  |
| 43. I feel abandoned by my friends or family |  |  |  |  |  |  |
| 44. I won't be bothered when other people talk about their children |  |  |  |  |  |  |
| 45. Because of infertility, I am worried that my spouse will be alienated from me |  |  |  |  |  |  |
| 46. When we talk about our fertility, I will comfort my spouse. |  |  |  |  |  |  |

#### Quality of life questionnaire

**Please select the answer that best reflects what you think and feel for each question (tick the box where you answer it). Connect your answers with your current thoughts and feelings. Some problems may be related to your private life, but they are necessary to fully measure all aspects of your life. Please choose the answer that is most appropriate to your current thoughts and feelings for each question.**

**A, how do you evaluate your health?**

**B. Are you satisfied with your quality of life?**

**□ very dissatisfied □ dissatisfied □ neither satisfied nor dissatisfied □ satisfied □ very satisfied**

**Q1. Is your attention and concentration affected by the idea of infertility?**

**□ Completely □ To a great extent □ Moderate □ Mild □ Not at all**

**Q2. Do you think you can't move on in life and achieve other life goals and plans because of the trouble of fertility problems?**

**□ Completely □ To a great extent □ Moderate □ Mild □ Not at all**

**Q3. Do you feel exhausted or exhausted because of the problem of fertility?**

**□ Completely □ To a great extent □ Moderate □ Mild □ Not at all**

**Q4. Do you think you have the ability to deal with your fertility problems?**

**□ Completely □ To a great extent □ Moderate □ Mild □ Not at all**

**Q5. Are you satisfied with your friends' support on your fertility?**

**□ very dissatisfied □ dissatisfied □ neither satisfied nor dissatisfied □ satisfied □ very satisfied**

***Q6. Are you satisfied with your sexual relationship, even though you are troubled by fertility problems?**

**□ very dissatisfied □ dissatisfied □ neither satisfied nor dissatisfied □ satisfied □ very satisfied**

**Q7. Does your fertility problem bring you jealousy or dissatisfaction?**

**□ Always □ Very often □ Very rarely □ Never**

**Q8. Do you feel sad and/or lost because you cannot have children or have more children?**

**□ Always □ Very often □ Very rarely □ Never**

**Q9. Are you wandering in the mood of hope and disappointment due to fertility problems?**

**□ Always □ Very often □ Very rarely □ Never**

**Q10. Are you isolated in social relationships because of fertility problems?**

**□ Always □ Very often □ Very rarely □ Never**

***Q11. Although you have fertility problems, are you still full of feelings with your partner?**

**□ Always □ Very often □ Very rarely □ Never**

**Q12. Does your birth interfere with your daily work or duties?**

**□ Always □ Very often □ Very rarely □ Never**

**Q13. Do you feel uncomfortable in social occasions such as holidays or celebrations due to birth problems?**

**□ Always □ Very often □ Very rarely □ Never**

**Q14. Do you think your family can understand everything you are going through?**

**□ Always □ Very often □ Very rarely □ Never**

***Q15. Does the fertility problem enhance your commitment to your partner?**

**□ Extreme □ Very □ Moderate □ Mild □ Not at all**

**Q16. Do you feel sad and depressed about your fertility problems?**

**□ Extreme □ Very □ Moderate □ Mild □ Not at all**

**Q17. Does your fertility problem make you feel inferior to people with children?**

**□ Extreme □ Very □ Moderate □ Mild □ Not at all**

**Q18. Do you suffer from fatigue due to birth problems?**

**□ Extreme □ Very □ Moderate □ Mild □ Not at all**

***Q19. Does the birth problem have a negative impact on your relationship with your partner?**

**□ Extreme □ Very □ Moderate □ Mild □ Not at all**

***Q20. Do you think it is difficult to communicate with your partner about your feelings about infertility?**

**□ Extreme □ Very □ Moderate □ Mild □ Not at all**

***Q21. Although you have fertility problems, are you satisfied with your relationship with your partner?**

**□ Extreme □ Very □ Moderate □ Mild □ Not at all**

**Q22. Do you feel that there is pressure from the society to have more children?**

**□ Extreme □ Very □ Moderate □ Mild □ Not at all**

**Q23. Does your fertility problem make you angry?**

**□ Extreme □ Very □ Moderate □ Mild □ Not at all**

**Q24. Do you feel pain and discomfort because of your birth problems?**

**□ Extreme □ Very □ Moderate □ Mild □ Not at all**

#### State-Trait Anxiety Inventory(STAI)

**Please select the answer that best reflects what you think and feel for each question (tick the box where you answer it). Connect your answers with your current thoughts and feelings. Some problems may be related to your private life, but they are necessary to fully measure all aspects of your life. Please choose the answer that is most appropriate to your current thoughts and feelings for each question.**

**T1. Does the treatment of infertility have a negative impact on your mood?**

**□ Always □ Very often □ Very rarely □ Never**

**T2. Can you get the infertility medical service you want?**

**□ Always □ Very often □ Very rarely □ Never**

**T3. How much trouble do you feel about your infertility treatment program and/or drug treatment?**

**□ Extreme □ Very □ Moderate □ Mild □ Not at all**

**T4. Are you worried about the impact of treatment on your daily life and work activities?**

**□ Extreme □ Very □ Moderate □ Mild □ Not at all**

**T5. Do you feel that the medical staff who treat infertility can understand everything you have experienced?**

**□ Extreme □ Very □ Moderate □ Mild □ Not at all**

**T6. Are you worried about the side effects of infertility drugs and treatment?**

**□ Extreme □ Very □ Moderate □ Mild □ Not at all**

**T7. Are you satisfied with the quality of service to solve your emotional needs?**

**□ very dissatisfied □ dissatisfied □ neither satisfied nor dissatisfied □ satisfied □ very satisfied**

**T8. How would you evaluate the surgery and/or medical treatment you received?**

**□ very dissatisfied □ dissatisfied □ neither satisfied nor dissatisfied □ satisfied □ very satisfied**

**T9. How will you evaluate the quality of the information about drugs, surgery and/or medical treatment you received?**

**□ very dissatisfied □ dissatisfied □ neither satisfied nor dissatisfied □ satisfied □ very satisfied**

**T10. Are you satisfied with your contact with the medical staff who treat infertility?**

**□ very dissatisfied □ dissatisfied □ neither satisfied nor dissatisfied □ satisfied □ very satisfied**

*Thank you very much for your patience and participation!*
